# The efficacy of a multi-strategy choice architecture intervention on improving the nutritional quality of high school students’ lunch purchases from online canteens (Click & Crunch High Schools): a cluster randomized controlled trial

**DOI:** 10.1186/s12966-022-01362-5

**Published:** 2022-09-14

**Authors:** Tessa Delaney, Sze Lin Yoong, Hannah Lamont, Christophe Lecathelinais, Luke Wolfenden, Tara Clinton-McHarg, Rachel Sutherland, Rebecca Wyse

**Affiliations:** 1 Hunter New England Population Health, Wallsend, NSW 2287 Australia; 2 School of Medicine and Public Health, The University of Newcastle, Callaghan, NSW 2308 Australia; 3 Hunter Medical Research Institute, Newcastle, NSW 2300 Australia; 4 Priority Research Centre for Health Behavior, The University of Newcastle, Callaghan, NSW 2308 Australia; 5 School of Health Sciences, Swinburne University of Technology, Hawthorn, VIC 3122 Australia

**Keywords:** Randomized controlled trial, Nudge, Choice architecture, Intervention, Web-based ordering systems, Digital intervention, Schools, Lunch, Canteen, Menu labeling

## Abstract

**Background:**

High school canteens are an ideal setting for public health nutrition intervention, and choice architecture strategies that facilitate the purchase of healthier foods and beverages from school canteens are recommended by the World Health Organization. The rapid uptake of online lunch ordering within school canteens provides a unique opportunity to implement choice architecture strategies that support healthier food choices with high fidelity. Despite this, no trial has tested the efficacy of choice architecture strategies within an online lunch ordering system on improving the nutritional quality of high school student lunch purchases. The objective of this study was to assess the impact of embedding choice architecture strategies into an online lunch ordering system on the nutritional quality of the school canteen lunch purchases of high school students (aged 12–19 years).

**Methods:**

A cluster randomized controlled trial was conducted with nine high schools in one Australian state. Schools were randomized to receive either a 2-month choice architecture intervention (involving menu labelling, prompts, item positioning, and feedback), or usual online ordering. Nutrient quality of online canteen lunch purchases was assessed using routine data collected by the online ordering system. Primary outcomes were the proportion of ‘Everyday’, ‘Occasional’, and ‘Should not be sold’ items purchased, categorized using the state healthy canteen policy. Secondary outcomes were the mean energy, saturated fat, sugar, and sodium content of purchases and the mean weekly revenue from online lunch orders. Linear mixed models were analyzed to assess outcomes.

**Results:**

Analysis of the student cohort (Intervention: 4 schools, 656 students; Control*:* 5 schools, 675 students) showed significant between group differences over time for the intervention group for the mean percentage of online lunch items per student that were ‘Everyday’ (+ 5.5%; *P* < 0.001) and ‘Should not be sold’ (− 4.4%; *P* < 0.001). There were no between group differences over time in the mean percentage of online lunch items that were ‘Occasional’; the average energy, saturated fat, sugar, or sodium content of lunch orders. There was also no difference in mean weekly revenue from high school student online lunch orders (*P* = 0.23).

**Conclusions:**

These findings suggest that a low intensity, choice architecture intervention embedded within an online ordering system can increase the purchase of healthier food items for high school students in one Australian state without any adverse impact on canteen revenue.

**Trial registration:**

This trial was prospectively registered on Open Science Framework on 23rd October 2020 as osf.io/h8zfr.

## Background

Unhealthy dietary patterns, including excess intake of energy, saturated fat, sugar and salt, are a leading cause of disease burden [1]. Whilst population dietary guidelines exist to support healthy eating behaviors, over 40% of adolescents’ (aged 14–18 years) daily energy intake comes from ‘discretionary foods’ that contribute to excess energy, saturated fat, salt and sugar [2–4]. These trends worsen over time as children mature through to adolescence [5]. For example, Australian population surveys have found that discretionary food consumption increases from 4.8 serves per day in children aged 4–8 years to 6.8 serves per day in adolescents aged 14–18 years [4]. As dietary patterns in adolescence carry through to adulthood and are associated with future risk of chronic diseases [6, 7], adolescence is a key period for public health nutrition intervention [8].

Schools are a recommended setting for the delivery of public health nutrition programs [9], as they provide centralized access and universal reach to students, and up to 40% of student dietary intake is consumed during school hours [10]. Schools are also a significant food provider [11]. For example, in Australia, over 60% of high school students purchase foods frequently from a school canteen (≥1 times/week) [11]. The majority of foods purchased from high school canteens, however, are typically high in energy, fat, salt or sugar [12] and the frequency of canteen use has been associated with higher adolescent weight to height ratio and cardio-metabolic risk [13]. As such, interventions to improve the nutritional quality of foods purchased by students from high school canteens are warranted to address the deterioration in dietary intake that occurs in adolescence.

To improve adolescent nutrition, Australian governments like those globally have introduced policies which aim to influence the relative availability of healthier foods from school canteens [14]. For example, in New South Wales (NSW) Australia, the ‘NSW Healthy School Canteen Strategy’ specifies that canteen menus must have at least 75% ‘Everyday’ items (healthy foods consistent with Dietary Guidelines), a maximum of 25% ‘Occasional’ items (those typically higher in energy, saturated fat, salt or sugar) and no ‘Should not be sold’ items (e.g. sugary drinks or items that do not meet a minimum guideline criteria) on the menu. Whilst the strategy is mandatory for all government schools, just 2% of high schools in most Australian jurisdictions implement such policies [14, 15], limiting their capacity to benefit child nutrition.

In order to maximize the impact of canteen-based interventions on adolescent diet, other environmental strategies that encourage healthier canteen purchasing are warranted. Choice architecture strategies that nudge people towards healthier food choices through small modifications to the food environment are recommended by the World Health Organization and have shown promise in school food settings for children and adolescents [16–18]. For example, a narrative review of 29 studies (including 7 in high schools) that assessed the impact of choice architecture interventions in school food settings found a positive association between strategies such as menu labelling, food placement and provision of prompts, and students’ selection of healthier foods [16]. Of these, 7 of the studies were undertaken in high schools and showed favorable effects of interventions on healthier food purchases. However, the majority (*n* = 6) of included high school studies were conducted in the United States and were non-randomized trials (*n* = 4). While such findings suggest that choice architecture strategies may encourage healthier purchasing among high school students, conducting randomized trials to examine the impact of such strategies in high schools that have different food service systems to the US (e.g. canteens that allow students to view a menu and either pre-order or purchase lunch, snacks or drinks over the counter compared to cafeterias which allow students to select lunch, side and drink options from a buffet) are warranted to address the existing evidence gaps.

Online school canteens allow users to access a menu, order and pay for menu items via the web or a mobile app, and are common in Australia and New Zealand [19]. They provide appealing infrastructure to embed choice architecture strategies targeting the selection of healthier foods for high school students. Importantly, given the wide reach of these systems, choice architecture strategies can be delivered efficiently at scale, and once such strategies have been applied to online systems, they have the potential to be maintained with high fidelity. Previous research targeting parent selection of food for primary school students has also demonstrated that choice architecture interventions delivered via online canteens are effective in improving the purchase of healthier menu items by up to 22% (*P* < 0.001) and decreasing the purchase of less healthy foods by up to 8% (*P* < 0.001) [19, 20]. As high school students have more autonomy over their food decisions relative to primary school students and there are differences in the barriers and enablers to healthy food choices between children and adolescents [21], the effects of such online canteen interventions delivered in primary schools may not generalize to high school students, and research is required to test these assumptions.

### Aim

The primary aims of the study were to assess the efficacy of embedding a choice architecture intervention (i.e. ‘*Click & Crunch High Schools’*) into an online canteen ordering system on the proportion of healthy (‘Everyday’) and less healthy (‘Occasional’ or ‘Should not be sold’) online lunch items purchased by high school students. The secondary aims were to assess the efficacy of *Click & Crunch High Schools* on the average energy, saturated fat, sugar and sodium content of high school student online lunch orders and the weekly revenue from online lunch orders.

## Methods

### Study design

This cohort study employed a parallel group, cluster randomized trial design. High schools located in New South Wales Australia, that were using an existing online canteen hosted by an online canteen provider (Flexischools) were randomized in a 1:1 ratio to receive either a 2-month multi-strategy behavioral intervention embedded in their online canteen or a usual practice control (the standard online canteen).

### Ethics and trial registry

The study was approved by the University of Newcastle Human Research Ethics Committee (H-2017–0402), New South Wales Department of Education and School Communities (SERAP 2018065) and relevant Catholic School Dioceses. The trial was prospectively registered on Open Science Framework on 23rd October 2020 [22] and is reported in accordance with the Consolidated Standards of Reporting Trials (CONSORT) extension for clustered Randomized Controlled Trials [23].

### Setting

The study took place in the state of New South Wales (NSW), Australia, a geographically and socio-economically diverse region with 827 schools enrolling high school students [24] and approximately 466,000 high school aged children (12–19 years) [25]. Flexischools is the leading provider of online canteens in Australia with the largest reach to over 1350 schools nationally and processing over 13 million lunch orders per annum (internal communication Flexischools). Flexischools, as with most online canteen providers, require users to download an app (usually to their mobile phone) to enable students to select and pay for their lunches.

### Participants

#### Schools

All government and non-government (independent or catholic) schools in NSW that: catered for high school students (including combined schools that catered for students from grades K-12); and used an online canteen provided by Flexischools, were eligible to participate (*n* = 134). Schools that had already participated in an ‘online canteen’ research program conducted by the team (*n* = 6) and catholic schools where ethical approval was not obtained by the diocese in time were also ineligible (*n* = 18). A post hoc exclusion criteria was added for schools that already had > 50% of their online menu labelled according to the NSW Healthy School Canteen Strategy due to the limited scope of applying labelling strategies as part of the intervention (*n* = 1 school).

#### Students

Any high school student from Grade 7 through to Grade 11 who placed an online order during the 2-month baseline data collection period (Term 4, Week 3–10, October to December, 2020) was eligible for inclusion in the trial. Students who were in Grade 12 during the baseline data collection period were excluded as they would have left the school prior to the intervention period (Term 1, Week 3–10, February to April, 2021). Furthermore, students with recurring lunch orders that were placed prior to the intervention period were excluded due to these participants not being exposed to the intervention strategies.

### Recruitment

All 134 schools in the sampling frame were approached from October 2020 to January 2021 (approximately 12 ‘school’ weeks) via mail and telephone with recruitment occurring until the required sample of schools consented to participate (*n* = 10). A recruitment manager oversaw recruitment of schools into the trial and monitored consent rates. Approximately 1 to 2 weeks after study invitations were posted to schools, a member of the research team telephoned the canteen manager to provide an overview of the study, confirm interest and answer any enquiries. Consent for study participation and access to canteen purchasing data was sought from the school Principal.

### Sample size

Assuming that 222 students per school placed at least one online lunch order over the data collection period (Flexischools, personal communication 2020), and assuming a standard deviation of 31% on the mean percentage of ‘Everyday’ items purchased by students [26], and an intra-class correlation coefficient of 0.05, the participation of 5 schools per arm would enable a mean detectable between group difference of 13% of ‘Everyday’ items, with 80% power and an alpha of 0.05.

### Randomization and blinding

Following school recruitment, schools were block randomized by an independent statistician using a random number function in Microsoft Excel®. Block randomization occurred at the unit of the school in randomly sequenced blocks of two and four. Given evidence that there are differences in healthy food availability between government and non-government schools (Catholic and Independent) [27], the randomization was stratified by school sector (government vs non-government). Due to the nature of the intervention, schools were not blinded to their group allocation, however the intervention was centrally applied, the online menu was only accessible to users (i.e. students) at individual schools and data was centrally collected by the online provider which prevented potential intervention contamination between groups.

### Intervention

#### Intervention development

The ‘*Click & Crunch High Schools’* intervention sought to encourage the purchase of healthier (i.e. ‘Everyday’) items from the school’s online canteen menu. The intervention was based on the principles of choice architecture which aim to structure environments in ways that cue desirable and automatic health related behaviors [28] such as healthier item selection from a menu. Choice architecture strategies may include information provision such as: adding nutrition labels at the point of purchase; altering the position of healthier items to make them more prominent and easier to access; and using prompts or automatic defaults to ‘nudge’ consumers towards healthier choices [28]. The selection of intervention strategies was developed in consultation with a multidisciplinary team including health behavior practitioners, dietitians, and the online canteen provider. Strategies were included if they were i) shown to be effective in improving healthier food purchases in school food settings [16, 20, 29]; ii) were considered appropriate and acceptable to principals [30] and; iii) were considered feasible for the online canteen provider to operationalize.

#### Intervention strategies

**Fig. 1:**
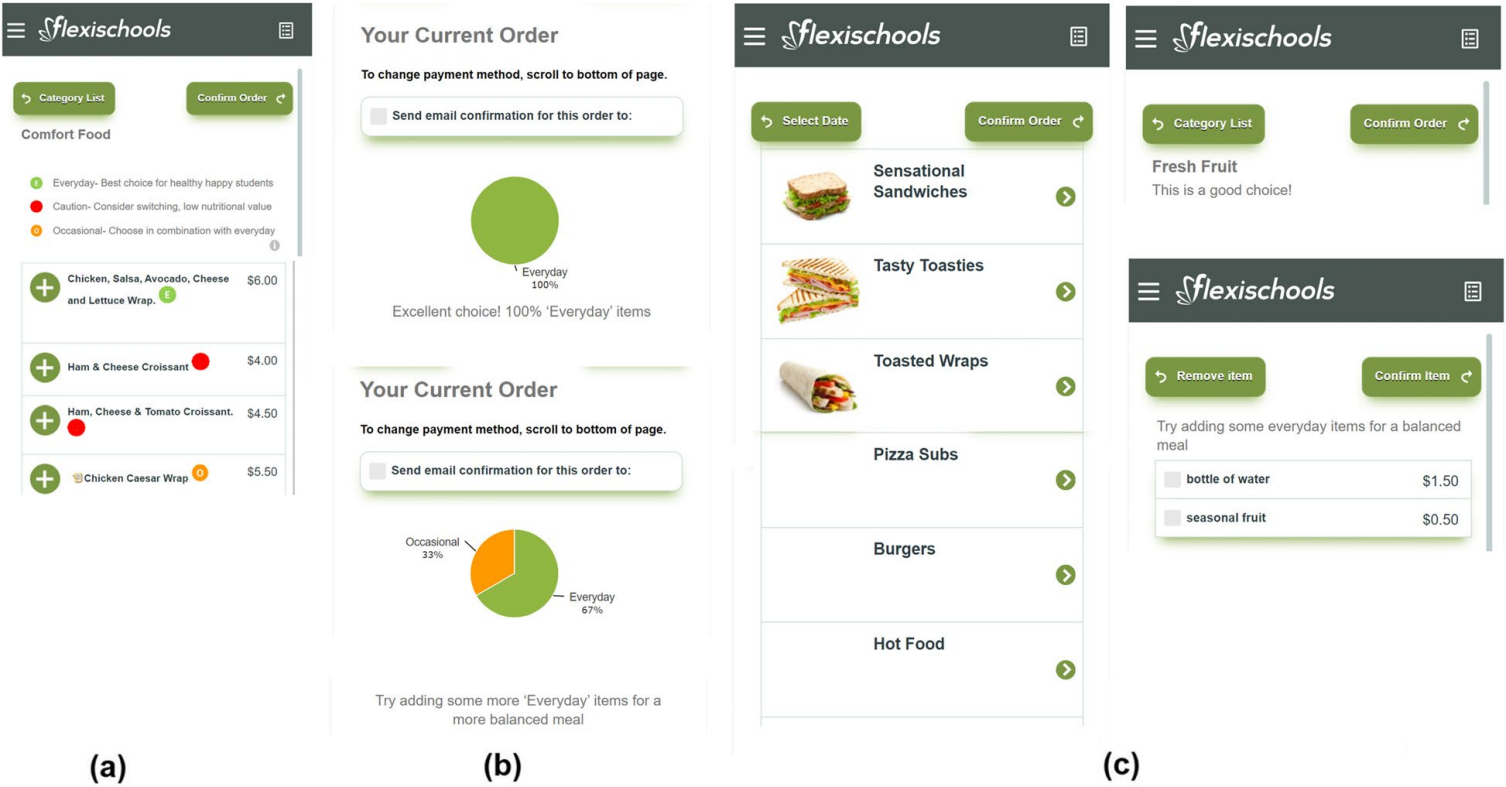
Screenshots from the Online Canteen Ordering System showing: **a** Menu labelling and positioning strategy - ‘Everyday’ first, ‘Caution’ (‘Should not be sold’) middle, ‘Occasional’ last; **b** Feedback strategy – pie chart displaying the proportion of ‘Everyday’, ‘Occasional’ and ‘Caution’ (‘Should not be sold’) items contained within the order plus a tailored message; **c** Prompts – Attractive image positioned next to healthy food categories; positive text prompts (“This is a good choice”) present for healthy food categories; and healthy ‘add-ons’ for ‘Occasional’ or ‘Caution’ (‘Should not be sold’) hot food items

**Fig. 2:**
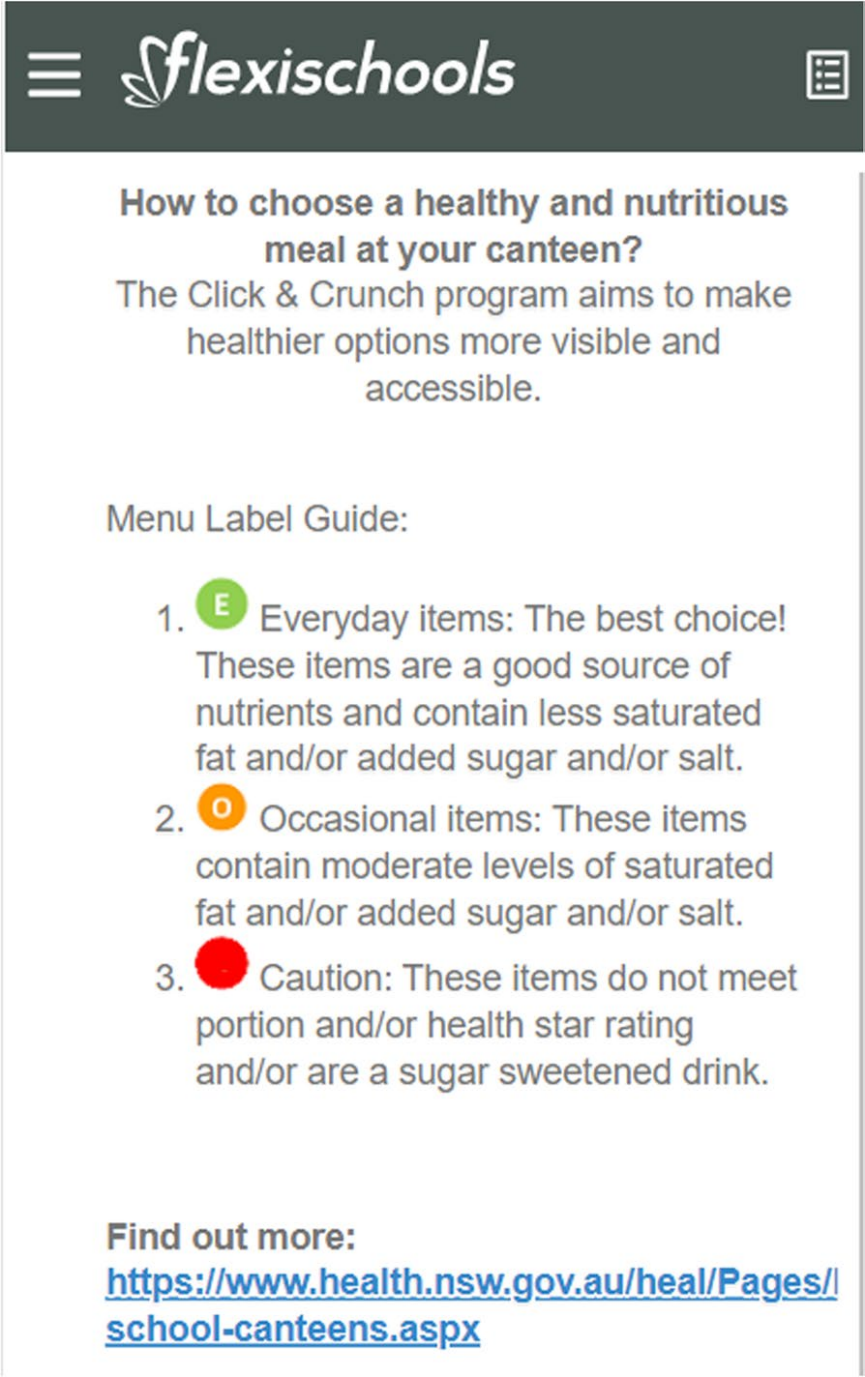
Detailed description of the labels and information about the NSW canteen strategy

All users of the online canteen at intervention schools had access to the ‘Click & Crunch High Schools’ intervention which was integrated into the school’s existing online canteen for 2 months (February to April 2021). Specifically, the ‘Click & Crunch High Schools’ intervention involved the following strategies (see Fig. 1) displayed to users of the program and at the point of purchase:

i) **Menu labelling:** Each item on the online menu was labelled with a colored symbol. The labelling system was based on the NSW Healthy School Canteen Strategy [31] and on previous evidence suggesting that traffic light labelling systems are effective in influencing the purchase of healthier menu items [20, 32]. Small symbols appeared next to each menu item; a green circle for ‘Everyday’ foods; an amber circle for ‘Occasional’ foods; and a red symbol for ‘Should not be sold’ (‘Caution’) foods. Within each menu category a ‘menu label key’ explaining the labels appeared at the top of the page (e.g. ‘Everyday – best choice for healthy happy students’; ‘Occasional – choose in combination with everyday’; ‘Caution – consider switching, low nutritional value’). A detailed description of the labels and information about the NSW Canteen Strategy were also able to be accessed by selecting the information tab at the bottom right-hand corner of the ‘menu label key’ (see Fig. 2).
ii) **Positioning:** Menu items were ordered to give healthier items positions of greatest prominence. This occurred at the food category level (e.g. drinks) and at the item level (e.g. plain milk). The healthiest food categories (e.g. fruit, salad, sandwiches) and items within categories (e.g. those that were labelled ‘Everyday’) were positioned first. Where there were multiple flavors of an ‘Occasional’ or ‘Should not be sold’ food (e.g. multiple flavors of chips), users were required to first ‘click’ on the item before the full list of flavors were displayed.
iii) **Feedback:** Prior to each lunch order being confirmed within the online ordering system, the user received feedback summarizing the nutritional content of the order. This was displayed using: i) a pie chart showing the proportion of ‘Everyday’, ‘Occasional’ and ‘Should not be sold’ items within the order; and ii) a tailored message based on the proportion of ‘Everyday’ items in the order as follows:

- < 99% ‘Everyday’ items: *“Try adding some ‘Everyday’ items for a more balanced meal”.*
- 100% ‘Everyday’ items: *“Excellent choice! 100% ‘Everyday’ items”.*

iv) **Prompts:** When a user selected an ‘Occasional’ or ‘Should not be sold’ hot food item, the user received a prompt to include a fruit or vegetable snack and water. Due to programming limitations, this strategy was applied to all hot food items typically sold as a single item (e.g. pie, burger) rather than items that were typically purchased in multiples (e.g. individual chicken nuggets). Additionally, healthy food categories (e.g. fruit, salads, sandwiches) also had a positive purchase prompt (e.g. “This is a good choice”) and an attractive image representing the food category applied immediately adjacent to the category name.

The labels and prompts were manually added to the online menus in intervention schools by the online canteen provider (based on a template provided by the research team). Once the online menus had been labelled, the positioning and feedback strategies were automatically applied to the menus. For example, when an ‘Everyday’ label was manually applied to a menu item, it would automatically position the item to appear first within the category list.

#### Supplementary support strategy

To facilitate canteen managers’ understanding of the labelling system and the labels that were applied to their online menu, each canteen manager in the intervention group received one ‘menu feedback report’. This feedback report provided an outline of how each menu item was labelled according to the NSW Healthy School Canteen Strategy (‘Everyday’, ‘Occasional’ and ‘Should not be sold’). The feedback report also included graphical feedback comparing the online canteen menu to the recommendations of the NSW Healthy School Canteen Strategy (i.e. ‘Everyday’ foods should comprise at least 75% of the menu, and ‘Should not be sold’ foods should be removed from the menu) and provided suggestions on how to improve the relative availability of ‘Everyday’ items.

#### Intervention Fidelity

Immediately after the intervention was applied (Week 3, Term 1, 2021) and mid-way through the intervention period (Week 5, Term 2, 2021) a member of the research team monitored each school’s online canteen menu via the provider’s website to ensure that all items (including any new items) were correctly classified, and the intervention strategies were applied accordingly. If the research team identified an unlabeled or incorrectly labeled item during these checks, the online canteen provider was notified and instructed to apply the intervention strategies as outlined in Fig. 1.

#### Control group

Control schools received no change to their online canteen menu, nor did they receive a ‘menu feedback report’ from the research team.

### Measures and data collection

Student purchasing data that was automatically collected by Flexischools was used to evaluate the primary and secondary trial outcomes. Trial outcomes used data from all online lunch purchases made by eligible students during the 2-month baseline and 2-month follow up periods. The baseline period was from week 3–10 of Term 4, 2020 (October to December), and the follow up period was immediately following intervention commencement from week 3–10 of Term 1, 2021 (February to April).

#### Characteristics of schools

School characteristics, such as the number of enrolments, year range, sector (government vs non-government), school type (combined schools that enroll both primary and high school students vs high school only) and postcode were obtained from the government ‘MySchool’ website [33]. For combined schools, high school student enrolment numbers were collected directly from the school as this data was not available on the ‘My School’ website. The number of canteen operating days per week, frequency of use and student grade were obtained from the student purchasing data supplied by Flexischools.

#### Usage rates

To describe the proportion of high school students that use online canteens, the number of online canteen users per school (from grades 7-12 in Term 1, 2021) was obtained from the purchase data supplied by Flexischools and divided by the number of high school enrolments collected via the ‘MySchool’ website for that school. These proportions were then averaged across all nine schools participating in the trial.

#### Primary outcomes

The primary outcomes were the mean percentage of all online lunch items purchased per student over the 2-month data collection periods that were classified as i) ‘Everyday’, ii) ‘Occasional’ and iii) ‘Should not be sold’ according to the NSW Healthy School Canteen Strategy [31]. After recruitment into the study, a research dietitian obtained a copy of the school’s online menu via Flexischools. In order for each purchased item to be classified against the strategy, detailed menu item information (brand, product name, serve size, flavor or recipe) was collected from each canteen manager over the phone or via email, using previously established data collection procedures [20, 34]. After the contact with the canteen manager, each menu item was classified by the dietitian. Following the supply of student purchasing data by Flexischools, a statistician then applied the menu item classification (e.g. ‘Everyday’) to the purchasing data (e.g. Salad = ‘Everyday’).

#### Secondary outcomes

##### The average energy, saturated fat, sugar and sodium content of students’ online lunch orders

As per previously established procedures [20, 34] the research dietitian obtained the nutritional profile (energy, saturated fat, sodium and sugar content per serve) of packaged products by matching the ‘brand’, ‘product name’, ‘serve size’ and ‘flavor’ (collected via phone call with the canteen manager) to a database containing over 2000 commonly stocked canteen products developed over 10 years by the research team [34]; if the product was not present in the database the dietitian used the following sources (in the following order) to obtain the nutritional profile: i) The FoodSwitch website/app [35], ii) an online search for the nutrient information panel, and iii) contacting the manufacturer directly. For canteen-made foods (e.g. ‘homemade muffins’), recipes were obtained from the canteen manager during the initial phone call and analyzed using the FoodWorks nutrition analysis software (Version 9). As per procedures described above, the nutritional profile of each product was then applied by the statistician to the student purchasing data supplied by Flexischools.

##### Weekly revenue from online orders

To assess any potentially adverse outcome of the intervention (e.g. reductions in revenue from applying the intervention), purchase data that was automatically collected by Flexischools was used to calculate the mean weekly revenue from all high school student online lunch orders for the weeks that the canteen was operational at baseline and follow-up.

#### Other data

##### Availability of Menu Items (Pre/Post Intervention)

To describe any changes in the availability of online menu items that resulted from the intervention, the proportion of items that were: ‘Everyday’, ‘Occasional’ and ‘Should not be sold’ on the online menu were assessed at baseline and follow-up based on the dietitian menu assessment procedures described above.

### Analysis

The analysis was conducted using an intention-to-treat approach whereby all student orders and schools were analyzed based on the groups to which they were originally allocated. Primary outcomes and secondary nutrition outcomes and subgroup analyses included data from all students that had baseline purchasing data. The school revenue outcome included all available data including from students without baseline purchasing data.

For each student, the primary trial outcomes were calculated by tallying the ‘total number of individual lunch items’ purchased over each of the two 2-month data collection periods (October – December 2020 and February – April 2021), and assigning scores of 0–100 corresponding to the percentage of those items that were classified as ‘Everyday’, ‘Occasional’ and ‘Should not be sold’. For example, if a student purchased 20 items over a data collection period and 10 were ‘Everyday’, a score of 50 would be assigned to represent 50% of items purchased that were classified as ‘Everyday’. These scores were then averaged across all students within the intervention and control groups. Three separate linear mixed models were analyzed to assess the primary trial outcomes. Each model compared intervention and control groups over time by including a group by time interaction fixed effect. All models included a random intercept for school (to account for potential school level clustering), a nested random intercept and random time effect for students (to account for repeated measurements between time points), and fixed effects for Socio-Economic Indexes for Australia (SEIFA) [36] and school sector.

All secondary trial outcomes, including differences in the i) nutritional content (average energy, saturated fat, sodium and sugar content) of all online lunch orders placed by students and ii) average weekly revenue that the canteen was operational were assessed using linear mixed models with a group by time interaction fixed effect. All models included a random intercept for school (to account for potential school level clustering) and fixed effects for sector and SEIFA. For secondary nutrition outcomes, a nested random intercept and random time effect for students (to account for repeated measurements within and between time points) were also included.

Subgroup analyses were conducted on the primary outcome in relation to ‘Everyday’ items, i.e. students’ mean percentage of ‘Everyday’ items and; i) student grade (grade 7 to 9 vs grade 10 to 11); ii) frequency of online canteen ordering (‘low’ < 1 order/week on average vs ‘high’ ≥ 1 order/week on average) and iii) school sector (government vs non-government). This was assessed as per the primary outcomes with the additional inclusion of a 3-way interaction term (group by time by subgroup) into the models.

A mixed logistic regression model, including a school random effect, was used to investigate whether being lost to follow-up was significantly associated with group (intervention vs control). Separate mixed logistic regression models were then used to assess whether that association differed on baseline characteristics (student grade; frequency of online canteen use) or nutrition outcomes, by adding an interaction fixed effect for each model.

Descriptive statistics were used to describe school characteristics, menu item availability and usage rates. All statistical analyses were performed in SAS version 9.3.

## Results

**Fig. 3:**
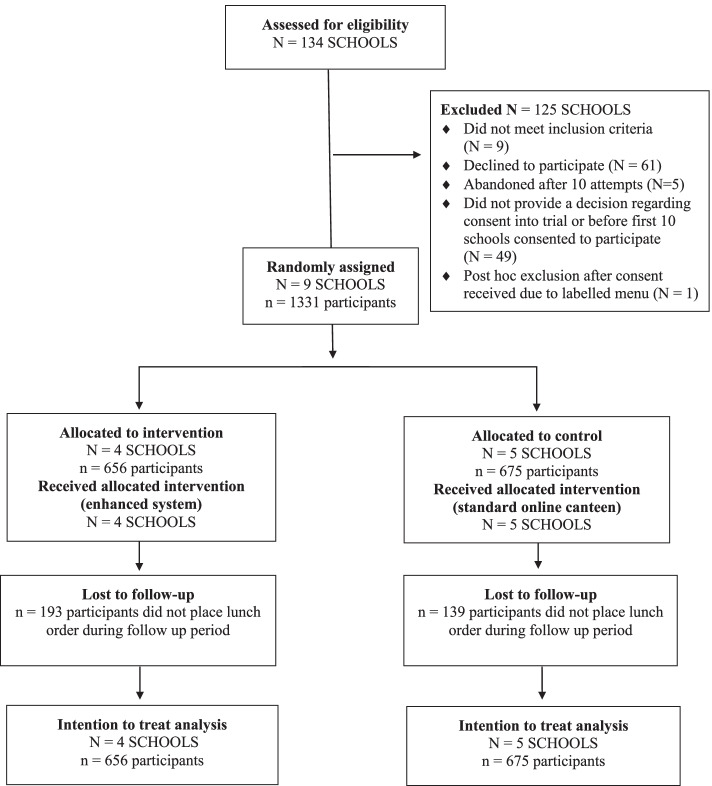
CONSORT flow diagram of participants through the trial and analysed for the primary outcome

Schools and participant flow through the trial can be seen in the CONSORT flow diagram (Fig. 3). After 12 weeks of recruitment, 9 eligible schools consented to participate and were randomized into the study, with 4 schools allocated to the intervention group and 5 to the control group. As shown in Fig. 3, 1331 students participated at baseline (656 intervention & 675 control) and 999 students (463 intervention & 536 control) at follow up. Participants in the intervention group were more likely to be lost to follow up than in the control group (intervention: 29.4%; control: 20.6%; *P* = 0.025), however there were no significant differences between the two groups being lost to follow-up based on student grade; frequency of online canteen use; baseline nutrients (e.g. mean energy, saturated fat, sugar or sodium) or; ‘Everyday’ or ‘Should not be sold’ item classification. There were however higher odds of being lost to follow-up for participants in the intervention group compared to the control group as the proportion of ‘Occasional’ items purchased increased (OR = 1.10, [95% CI 1.01, 1.19]; *P* = .03). The baseline characteristics between groups are described in the tables below (see Table 1 and Table 2). At baseline, the control group had higher student enrolments (mean 800 ± SD 318) compared to the intervention group (mean 496 ± SD 226). All other baseline characteristics were relatively similar between intervention and control groups. In total, 16,829 lunch orders and 28,710 menu items were included in the analysis of study outcomes.

**Table 1:** Characteristics of the sample of 9 participating NSW schools

School & Canteen Characteristics	Intervention ***n*** = 4 schools	Control ***n*** = 5 schools
**School sector, n(%)**		
Government	1 (25%)	2 (40%)
Non-Government^a^	3 (75%)	3 (60%)
**School type, n(%)**		
Combined school (students aged 5–19 years)	3 (75%)	2 (40%)
High school (students aged 12–19 years)	1 (25%)	3 (60%)
**Number of enrolments**^**b**^**, mean(SD)**	496 (226)	800 (318)
**Socioeconomic status of school**^**c**^**, n(%)**		
Least advantaged	1 (25%)	2 (40%)
Most advantaged	3 (75%)	3 (60%)
**Canteen days of operation**^**d**^**, n(%)**		
5 days a week	4 (100%)	5 (100%)
**Canteen menu characteristics**^**e**^**, n(%)**		
≥ 75% Everyday items on menu	2 (50%)	2 (40%)
No ‘should not be sold’ items on menu	0 (0%)	0 (0%)
**Number of weekly online lunch orders per school**^**d**^**, mean(SD)**	141 (62)	135 (77)

^a^ Non-government schools were Catholic and Independent Schools

^b^ Based on publicly available school statistics (MySchool 2020)

^c^ Socio Economic Indexes for Australia 2016, based on postcode of school locality and dichotomised at the NSW median

^d^ Based on Flexischools purchasing data

^e^ As classified by a dietitian according to the NSW Healthy School Canteen Strategy

**Table 2:** Characteristics of the sample of 1331 participating students

User Characteristics^**a**^	Intervention ***N*** = 656 participants	Control ***N*** = 675 participants
**Grade of student at baseline, n(%)**		
Grade 7–9	503 (77%)	541 (80%)
Grade 10–11	153 (23%)	134 (20%)
**Frequency of use, n(%)**		
High users (≥1 order / week on average)	166 (25%)	219 (32%)
Low users (< 1 order / week on average)	490 (75%)	456 (68%)

^a^ Based on Flexischools purchasing data

### Usage rates

The average proportion of high school students using an online canteen (across all nine schools) was 29.1% (1740/5981, range 6.2–56.7%).

### Intervention Fidelity

The proportion of unlabeled or incorrectly labelled items across the 4 intervention schools was 0% (0/508 items, Week 3, Term 12,021) and 0.20% (1/508 items, Week 5, Term 12,021), indicating a high level of intervention fidelity.

### Primary outcomes

The results from the mixed model analysis indicate that there were significant between group differences over time favoring the intervention group for the mean percentage of all online lunch items per student that were Everyday (+ 5.5%; [95% CI 2.2, 8.9]; *P* < 0.001) and ‘Should not be sold’ (− 4.4%; [95% CI -7.0, − 1.8]; *P* < 0.001) (Table 3). There were no between group differences over time in the mean percent of lunch items per student classified as ‘Occasional (− 1.2%; [95% CI -4.3, 2.0]; *P* = 0.47).

**Table 3:** Differences in intervention and control groups over time for primary and secondary trial outcomes^a^

	Intervention		Control		Intervention vs Control^**a**^	
	Baseline Mean (SD) ***n*** = 656 students	Follow up Mean (SD) ***n*** = 463 students	Baseline Mean (SD) ***n*** = 675 students	Follow up Mean (SD) ***n*** = 536 students	Main analysis	
					Group by time differential effect (95% CI)	***P***
**Primary Outcomes**						
Mean % per student of lunch items that are ‘Everyday’	44.3 (34.3)	48.1 (32.2)	43.2 (36.3)	40.8 (35.2)	5.5 (2.2, 8.9)	0.001*
Mean % per student of lunch items that are ‘Occasional’	30.7 (30.8)	30.4 (29.9)	40.9 (35.7)	43.4 (35.0)	−1.2 (−4.3, 2.0)	0.47
Mean % per student of lunch items that are ‘Should not be sold’	25.0 (30.9)	21.5 (29.0)	16.0 (24.7)	15.9 (24.5)	−4.4 (−7.0, −1.8)	0.001*
**Secondary Outcomes**						
Mean energy (kilojoules) per student lunch order	2172.5 (976.9)	2214.6 (937.4)	1992.8 (793.1)	1944.5 (740.8)	−15.3 (−78.3, 47.7)	0.63
Mean saturated fat (grams) per student lunch order	7.3 (5.7)	7.3 (5.5)	5.7 (4.4)	5.3 (3.6)	−0.2 (− 0.5, 0.2)	0.42
Mean sugar (grams) per student lunch order	22.4 (22.8)	22.8 (22.3)	15.3 (16.2)	13.9 (14.8)	0.0 (−1.3, 1.4)	0.97
Mean sodium (milligrams) per student lunch order	778.9 (354.1)	808.5 (361.6)	808.3 (398.2)	791.1 (397.5)	24.5 (−3.9, 52.9)	0.09
Mean weekly revenue per school ($AUD)^b^	896.1 (449.9)	1057.0 (387.2)	769.6 (372.8)	1053.7 (486.6)	−117.1 (−328.9, 94.6)	0.23

^a ^Data were analyzed using linear mixed models and included a group by time interaction fixed effect, a random intercept for school, a nested random intercept and random time effect for students (to account for repeated measurements between time points), and fixed effects for SEIFA and school sector

* Denotes a statistically significant *P*-value of <.05

### Secondary outcomes

#### The average energy, saturated fat, sugar and sodium content of students’ online lunch orders

There were no between group differences over time in the average energy (− 15 kJ; [95% CI -78.3, 47.7]; *P =* 0.63), saturated fat (− 0.2 g; [95% CI -0.5, 0.2]; *P =* 0.42), sugar (0.0 g; [95% CI -1.3, 1.4]; *P =* 0.97) and sodium (+ 24.5 mg; [95% CI -3.9, 52.9]; *P =* 0.09) content of student online lunch orders.

#### Weekly revenue from online orders

There were no significant between group differences in the average weekly revenue from online lunch purchases over time (AUD $-117.10; [95% CI AUD $-328.9, 94.6]; *P* = 0.23).

### Subgroups

There were no differences in intervention effectiveness over time with respect to the mean percentage of all online lunch items per student that were ‘Everyday’ by the subgroups of student grade, frequency of online canteen ordering or school sector (see Table 4).

**Table 4:** Impact of the intervention on student’s mean percentage of ‘Everyday’ online lunch items: subgroup analysis^a^

Variable	Intervention		Control		Intervention vs Control^**a**^			
	Baseline Mean (SD) ***n*** = 656 students	Follow up Mean (SD) ***n*** = 463 students	Baseline Mean (SD) ***n*** = 675 students	Follow up Mean (SD) ***n*** = 536 students	Group by time differential Effect (95% CI)	***P***	Group by time by Subgroup differential effect (95% CI)	***P***
**Student grade**								
Grade 7–9	44.0 (33.8)	47.2 (32.1)	42.8 (36.1)	41.6 (36.0)	3.9 (0.2, 7.7)	0.04	–	–
Grade 10–11	45.4 (35.9)	51.2 (32.8)	44.6 (37.3)	37.1 (31.2)	11.7 (4.2, 19.1)	0.002	7.7 (−0.6, 16.1)	0.07
**Frequency of use**								
High users (≥1 order / week on average)	43.9 (29.7)	49.0 (32.4)	43.8 (30.9)	40.7 (32.3)	7.7 (2.5, 12.9)	0.004	3.88 (−2.92, 10.69)	0.26
Low users (< 1 order / week on average)	44.6 (36.7)	47.4 (32.2)	42.7 (39.2)	40.8 (37.2)	3.85 (−0.5, 8.2)	0.08	–	–
**School sector**								
Government	43.9 (36.5)	45.9 (33.1)	29.9 (30.1)	27.3 (27.8)	5.1 (−1.4, 11.7)	0.12	–	–
Non-Government	44.6 (33.2)	49.4 (31.7)	46.6 (37.0)	44.1 (36.0)	6.0 (2.0, 10.0)	0.003	0.9 (−6.8, 8.5)	0.82

^a^Data were analyzed using linear mixed models and included a group by time interaction fixed effect, a random intercept for school, a nested random intercept and random time effect for students (to account for repeated measurements between time points), and fixed effects for SEIFA and school sector

### Other data

#### Availability of menu items

At baseline and follow-up, no intervention or control schools had lunch menus that met the NSW Healthy School Canteen Strategy criteria of at least 75% ‘Everyday’ and 0% ‘Should not be sold’ items. At baseline, the mean availability of ‘Everyday’ (69.4 and 68.0%), ‘Occasional’ (14.9 and 20.0%) and ‘Should not be sold’ (15.7 and 12.0%) items were similar by intervention and control menus respectively. At follow up, the mean availability of ‘Everyday’ (72.0 and 67.0%); ‘Occasional’ (14.8 and 21.6%); and ‘Should not be sold’ items (13.2 and 11.5%) were similar by intervention and control menus respectively (no significance testing conducted).

## Discussion

### Principal results

To our knowledge, this is the first randomized controlled trial to assess the efficacy of a multi-strategy choice architecture intervention for improving the nutritional quality of high school students’ purchases from online canteens. The study found that over time, high school student lunch purchases in the intervention group contained a significantly higher average proportion of ‘Everyday’ items and lower proportion of ‘Should not be sold’ items than those in the control group (*P* < 0.001 respectively). Encouragingly, the intervention did not have any adverse impact on canteen revenue, a frequently reported barrier by canteen managers to the uptake and adherence of public health nutrition interventions in canteens. These findings suggest that a choice architecture intervention embedded within an online ordering system can improve the selection of healthier food options for high school students in one Australian state.

While small, the intervention effect, which was equivalent to a 5.5% increase in ‘Everyday’ items and a 4.4% reduction in ‘Should not be sold’ items per student, is likely beneficial from a population health perspective. For example, according to the NSW Canteen Strategy foods classified as ‘Everyday’ are defined as those aligned with core food groups identified in the Australian Dietary Guidelines (fruit, vegetables, dairy and/or alternatives, grains, lean meat and/or alternatives) and those classified as ‘Should Not Be Sold’ are ‘discretionary’ foods [31, 37]. Together these findings suggest that the intervention resulted in beneficial changes consistent with the recommendations in the Australian Dietary Guidelines (2013) which provides population dietary advice on maintaining good health and protection against chronic disease [37]. Furthermore, modelling studies have found that small cumulative changes in dietary intake from less healthy to healthier ‘everyday’ alternatives can result in population health benefits and cost savings [38, 39].

To our knowledge, no studies have tested the efficacy of delivering choice architecture strategies via web or digital modalities to improve the selection or purchase of healthier foods from high schools. A before and after study conducted in two New York high school lunch rooms using strategies directly applied in the physical food service environment including placement, prompts, and improved access to healthier foods, found that students selection of fruit and vegetables increased from 47 to 54% (*P* = .012) and 36 to 44% (*P* < .001) respectively after a 2 month intervention [40]. A non-randomized trial of multicomponent choice architecture intervention in two high school canteens in the UK found that student selection of designated items (items with a fruit, vegetable or salad component) increased significantly during the intervention (3%) and post intervention (2.2%) periods compared to baseline (1.4%) [41].

The trial findings and pattern of results are consistent with previous research conducted in primary school online canteens [20, 42]. For example, the Click & Crunch intervention in 17 NSW primary schools found a 9.8% increase in the purchase of ‘healthier’ foods and a 7.7% decrease in the purchase of ‘less healthy’ foods respectively. The differences in effect sizes could be due to a range of factors, including (but not limited to) different canteen operating models, increased availability of healthier options in primary schools and the differences in the primary target of the intervention (parents vs students) [43]. Whilst a smaller magnitude of effect was found with high school students, the intervention is likely to be particularly appealing to policy makers as public health nutrition interventions targeting school canteens often fail to translate between primary and high school settings [43, 44] due to differences in canteen operating models [45], concerns over profitability [46], who makes the purchasing decisions (parents or students) and the perceived differences in demand for menu items by different age groups [14, 46, 47].

For secondary outcomes, there were no between group differences over time in the average energy, saturated fat, sugar, or sodium content of lunch orders. This finding may be partially explained by the labelling system, the mechanism underpinning the intervention. The labelling system of the NSW Healthy School Canteen Strategy classifies menu items by their overall nutritional quality (‘Everyday’ vs ‘Occasional’) rather than by their individual nutrients (e.g. kilojoule labelling). Therefore, students would be made aware of a menu item’s overall nutritional quality, but not the specific contribution of the underlying nutrients such as the energy, sugar, sodium, or saturated fat. It is therefore possible for students to switch from categories of food (‘Should not be sold’ to ‘Occasional’) without observing differences in energy, sugar, sodium or saturated fat. For example, a ‘Should not be sold’ item such as confectionary in a serve size of 12.5 g may contain approximately 200 kJ, 12 g of sugar (94 g per 100 g), 0 g of saturated fat, 80 mg of sodium, and offers little nutritional value [35]. In contrast, a nutritious ‘Everyday’ item such as a green apple (166 g) contains approximately 340 kJ, 17 g of sugar (10 g per 100 g), and is a good source of fibre [48]. A popular ‘Occasional’ item such as commercial popcorn (25 g), may contain 445 kJ, 0.4 g of saturated fat, and 90 mg of sodium [49].

Finally, the study found no differences in intervention effectiveness over time by student grade (grade 7–9 vs 10–11), frequency of online canteen use (≥1 order/week vs < 1 order/week) or school sector (government vs non-government). This indicates that the intervention has a broad public benefit across varying social strata and subgroups, however it should be noted that the analysis was exploratory, potentially underpowered and the findings in relation to student age was borderline significant and may warrant further investigation.

### Strengths and limitations

The strengths of this study include the use of a randomized controlled trial design, the application of a state government labelling system, high intervention fidelity, use of objective and routinely collected purchasing data, and the inclusion of both government and non-government schools. The findings however should also be considered in the context of the study’s limitations. First, whilst NSW survey data suggests that at least 60% of high school students purchase their lunch at least weekly from a school canteen, our study found relatively low use of online canteens by high school students, with up to 32% ordering their lunch at least weekly from these systems. This may suggest that high school students use the school canteen for ‘spontaneous’ or over the counter purchases rather than for ordering their lunch. Therefore, the impact of this efficacious intervention may be enhanced by combining other strategies targeting the physical food environment of high school canteens. Second, the small number of schools (clusters) may have affected randomization and overall generalizability of findings. Encouragingly though, the study included data from 1331 students and the school and user characteristics were relatively similar between intervention and control groups. Third, the study was undertaken in one Australian jurisdiction and the generalizability of the findings may be limited to jurisdictions with similar school food service models (online lunch ordering) and/or menu labelling systems (traffic light labels). In addition to Australia, online lunch ordering is becoming increasingly common across schools in the USA [50] and United Kingdom [51] and future research should investigate the effectiveness of this intervention within these emerging settings. Furthermore, most Australian jurisdictions utilize traffic light labelling systems to classify canteen menu items as ‘healthier’ or ‘less healthy’ [14]. Nevertheless, the inclusion of more schools, across multiple state, national and international jurisdictions would have increased the generalizability of the findings. There was also greater attrition of participants from the intervention group than from the control group. Whilst there were very few differences in the characteristics of participants who were lost to follow up between the two groups, further research which qualitatively explores the acceptability of the intervention and barriers to online ordering uptake by high-school students and key stakeholders may be warranted. Additionally, the intervention was conducted over a relatively short timeframe (2 months) and future research which assesses the long-term effect of the intervention is required. Finally, despite relatively little investment required by schools to participate in the study, only 9 eligible schools provided consent with 46% of schools declining to participate. The study recruitment, which was undertaken in the last quarter of 2020, may have been adversely impacted by COVID-19 where some schools and canteens across NSW were impacted by temporary closures due to government issued ‘stay at home orders’ [52, 53]. Nevertheless, future research that involves a larger sample of high schools across multiple jurisdictions, is conducted post pandemic, over a longer timeframe and explores the acceptability to high school students and stakeholders is warranted.

## Conclusions

This study provides evidence supporting the efficacy of a behavioral intervention embedded in online canteens to improve the healthiness of student purchases from high school canteens in one Australian state. Given the evidence of its efficacy and ability to be implemented at high fidelity, and evidence of no adverse impact on canteen revenue, this behavioral intervention may represent an appealing option in isolation or in combination with broader public health nutrition strategies in the Australian high school setting. However, in order to explore the long-term effectiveness of the intervention and its effect at scale further research is warranted over a longer period of time and involving more high schools across multiple jurisdictions where online lunch ordering is utilized.

## Data Availability

The datasets used and/or analysed during the current study are available from the corresponding author on reasonable request.

## Abbreviations

NSW: New South Wales
SEIFA: Socio-Economic Indexes for Australia
AUD: Australian Dollar
